# Application of lateral supraclavicular incision in unilateral thyroid papillary carcinoma surgery

**DOI:** 10.1016/j.bjorl.2021.03.010

**Published:** 2021-04-20

**Authors:** Yan-Xin Ren, Jie Yang, Wei-Ze Sun, Yun Chen, Xi-Fang Wu, Ning Huang, Xiao-Jiang Li

**Affiliations:** a Kunming Medical University, Third Affiliated Hospital, Kunming, China; b Kunming Medical University, Third Affiliated Hospital, Department of Pathology, Kunming, China; c Kunming Medical University, Department of Pharmacology, Kunming, China

**Keywords:** Thyroidectomy, Surgical incision, Cosmetology

## Abstract

**Introduction:**

The standard approach to thyroidectomy is a collar incision via the anterior neck, and the neck scar has always been a source of worry for patients. Acceptable wound cosmetology has become a focus for thyroid surgeons.

**Objective:**

To verify the effectiveness and cosmetic results of thyroidectomy through a lateral supraclavicular incision.

**Methods:**

180 patients were randomly divided into two groups: a lateral supraclavicular approach and a conventional transcervical approach. The main outcomes included incision length, intraoperative blood loss, operative time, total drainage volume, hospitalization expense, early postoperative pain measured by visual analog scale, infection, and perceived cosmetic outcome.

**Results:**

There were no statistical significances between the two groups in terms of age, gender, nodule size, intraoperative blood loss, operative time, total drainage volume, hospital expense and postoperative complications, whereas there were significant differences in terms of incision length (5.2 ± 1.04 cm vs. 6.9 ± 1.14 cm, *p* < 0.05).

**Conclusions:**

The lateral supraclavicular incision is a safe and feasible approach for thyroidectomy. Compared with conventional approach, it provides a better cosmetic result.

## Highlights

• The lateral supraclavicular incision in unilateral thyroid carcinoma surgery provides satisfactory cosmetic outcomes.
• The lateral supraclavicular incision is a safe and practical approach for thyroidectomy.

## Introduction

Thyroid nodules are a common clinical problem, with an estimated prevalence based on palpation evaluation that ranges from 3% to 7%.^1^ Thyroid carcinoma, especially papillary thyroid carcinoma, is a well-differentiated, slow-growing tumor with a relatively good prognosis, but the incidence of thyroid carcinoma has been increasing.^2^, ^3^ At present, the effect of drug therapy is limited, so surgery is an important and effective method to treat thyroid carcinoma.^4^ The standard approach to thyroidectomy has been through a collar incision via the anterior neck, which was originally developed by Theodore Kocher in the late 19th century. In the past 20 years, alternative approaches have been described, including endoscopic (video-assisted) thyroidectomy, robotic thyroidectomy and transoral thyroidectomy, in order to avoid neck scar.^5^, ^6^ However, the strict indications, expensive equipment, and emerging complications restrict their application.

To get a better esthetic effect, Yang^7^ and Chen et al.^8^ modified the location of the incision by moving it to the supraclavicular cavity, so the incision can be hidden under a collar neck decoration. While, the technique was indicated in strictly benign thyroid nodules, the objective of this study was to assess efficacy and safety of unilateral thyroidectomy through a lateral supraclavicular approach in malignant thyroid nodules.

## Methods

From February 2019 to January 2020, 180 patients were assessed for eligibility after the research accepted the approval of the hospital's ethics committee (KY2020216). All patients had unilateral thyroid nodules and underwent fine needle aspiration biopsy (FNAB) in our department. Results showed that these were thyroid papillary carcinoma. The following patients were excluded from this study: (1) presence of evidence of the lateral neck lymph node metastasis by needle biopsy; (2) ultrasonic- suggested bilateral thyroid nodules which would suggest total thyroidectomy; (3) a history of previous neck surgery or head and neck irradiation; (4) presence of evidence of distant metastasis by ultrasonography and computerized tomography; (5) patients with known blood-clotting disorders; (6) a recent history of fever or infection; (7) pregnancy, severe chronic diseases; (8) the tumor was located behind the sternum. During the time of hospitalization, complete preoperative evaluations (biochemical assessment, laboratory thyroid function, ultrasonography, and computed tomography) were obtained and evaluated in all cases. All patients underwent preoperative laryngoscopy to assess vocal fold movement. Patients who agreed to the study criteria were randomly divided into two groups (LS group: lateral supraclavicular group and CT group: conventional transcervical group).

### Surgical procedure

**Figure 1:**
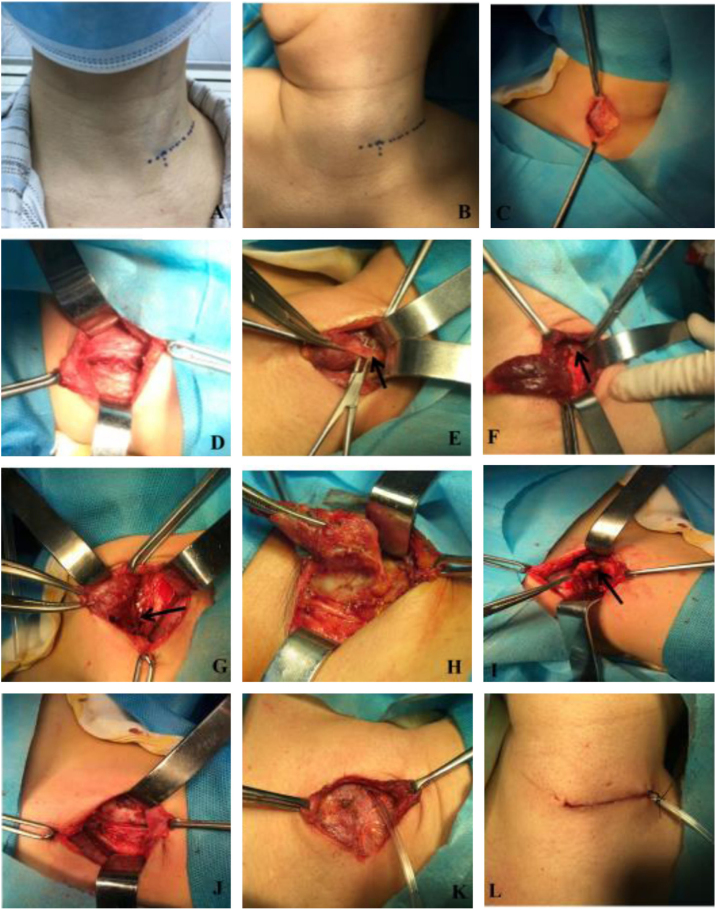
Surgical procedure. A, the patient was carefully marked for the incision location supraclavicularly and laterally to the neck along with the skin crease when sitting up; B, Patient was in the supine position with head to contralateral; C, The skin and platysma muscle was transected and subplatysmal flap was created; D, The strap muscles were cut longitudinally and retracted; E, The upper vascular pedicles. (→) was cut and ligatured; F, The upper parathyroid gland (→) was preserved with their blood supply intact; G, The recurrent laryngeal nerve (→) was dissected; H, The inferior vascular pedicles was cut and ligatured; I, The central lymph nodes (→) was dissected; J, The full view of operative cavity after irrigation; K, The strap muscles was reapproximated and a 10-gaged silicone tube was placed in the thyroid cavity; L, Incision was closed with subcutaneous absorbable surgical suture.

For the lateral supraclavicular group, a lateral supraclavicular collar incision was chosen. Patients were placed under general endotracheal anesthesia in the supine position on the operating table with head to contralateral; the incision was about 2 cm away from the sternoclavicular joint, 5–6 cm long, and parallel with the clavicle at the outer edge of the sternocleidomastoid (Fig. 1A–B). The platysma muscle was transected and subplatysmal flap was created. Then, the cervical fascia was incised just along the anterior border of the sternocleidomastoid to expose the strap muscles. The strap muscles were cut longitudinally and retracted laterally. Subsequently, the gland lobe was directly exposed just under the incision. This would facilitate the division and ligation of the middle thyroid vein, superior and inferior pole blood vessels. The ipsilateral thyroid lobe and the isthmus were then dissected off (Fig. 1C–H).

Next, ipsilateral prophylactic central neck dissection was performed routinely. The prelaryngeal, pretracheal and paratracheal lymph nodes were incised to expose the trachea down to the level of the brachiocephalic vessels inferiorly and the medial border of the carotid artery was dissected down to the prevertebral fascia. The superior limit of dissection was at the level of the cricoid cartilage. The recurrent laryngeal was freed from the fibrofatty tissue of the paratracheal space, allowing for lateral retraction of the recurrent laryngeal nerve away from the level VI lymph nodes. Care was taken to preserve the superior parathyroid on a vascularized pedicle, the inferior parathyroid gland was typically devascularized and removed for auto transplantation by injection technique^9^ (Fig. 1I–J).

At the end of the surgical procedure, saline was used to irrigate the remained cavity and hemostasis was assured. A 10 gauge silicone tube was placed in the thyroid cavity, the strap muscles and platysma muscle were reapproximated and the incision closed with subcutaneous absorbable surgical suture (Fig. 1K–L).

For the conventional transcervical group, an anterior median incision was adopted. A 5–7 cm long incision was made horizontally about one finger above the suprasternal notch. The platysmal muscle was transected, and a subplatysmal flp was created. The fascia over the strap muscles was incised in the midline, and the sternohyoid muscles are carefully dissected and retracted laterally to expose the underlying sternothyroid muscles and anterior surface of the thyroid gland. The sternothyroid muscles are divided near their upper end to gain exposure of the upper pole of the thyroid gland. The entire thyroid gland was exposed, and total lobectomy was conducted. Ipsilateral central neck dissection was conducted in similar fashion.

All surgical procedures were performed under general anesthesia by the same group of surgeons. The recurrent laryngeal nerve and parathyroid glands were routinely preserved with their blood supply intact.

### Observation index and statistical analysis

No patients in the lateral supraclavicular group required conversion to conventional surgery. The study included the measurement of incision length, intraoperative blood loss, operative time, total drainage volume, hospitalization expense, final pathologic findings, postoperative complications, and the clinical characteristics of the patients were collected and recorded into our database.

The postoperative pain and cosmetic satisfaction data were collected using a 10-point Visual Analog Scale (VAS) as follows: postoperative pain was assessed using VAS, which ranges from a score of 0 (painless) to 10 (the most severe pain).^10^ Cosmetic score was measured 3 months after surgery, the clinical evaluation divisions were as follows: 0 to 3 means fair cosmetic effects, 4 to 6 means moderate cosmetic effects, and 7 to 10 means good cosmetic effects, these scores were recorded.^11^

All the continuous variables were expressed as mean standard deviation. The statistical analysis was performed with the two-tailed Student's *t*-test for normally distributive data. A nonparametric test (Mann–Whitney test) was performed when distribution was non-normal. All values were expressed as mean ± SD for continuous variables and number (percentage) for categorical variables, statistics were calculated using SPSS 17.0 for Windows (SPSS, Inc, Chicago, IL).

## Results

In this study, 180 patients were enrolled in the two 2 groups equally. The epidemiological and clinicopathologic data of the two groups, such as age, sex, tumor size and body mass index(BMI) were similar, which are summarized in Table 1.

**Table 1:** Clinicopathological characteristics of each group.

Clinicopathologic features	LS group *N* = 90	CT group *N* = 90	*p*
*Age (y)*	38.9 ± 3.21	40.1 ± 2.54	0.754

*Gender*			
Male	35	41	0.861
Female	55	49	

*BMI*	23.4 ± 2.70	22.5 ± 1.92	0.647
*Lymphnode number*	6.61 ± 1.44	5.98 ± 1.62	0.251

*Lymph node metastasis*			
Yes	37	35	0.761
No	53	55	

*Tumor size (cm)*	2.35 ± 0.21	2.58 ± 0.31	0.527
*Blood loss (mL)*	14.92 ± 1.25	16.54 ± 1.33	0.395
*Operative time (min)*	82.30 ± 7.53	80.55 ± 6.94	0.447
*Drainage volume*	132.54 ± 18.72	145.68 ± 18.72	0.253
*Hospital expense (Thousand RMB)*	11.08 ± 2.71	10.94 ± 2.43	0.853
*Incision length (cm)*	5.23 ± 1.04	6.93 ± 1.18	***0.041***

LS, lateral supraclavicular; CT, conventional transcervica; BMI, body mass index.

### Surgical outcomes & pathologic results

All the intraoperative variables are shown in Table 1. There were no statistically significant differences in intraoperative blood loss, operative time, total drainage volume and hospitalization expense between the two groups (*p* > 0.05). The incision length was significantly different between the two groups: it was 5.2 ± 1.04 cm versus cm in the LS group and 6.9 ± 1.14 cm in the CT group (*p* < 0.05). The number of lymph nodes and lymph node metastasis rates in the LS group were higher than that in the CT group (6.61 ± 1.44 vs. 5.98 ± 1.62; 41.1% vs. 38.9%), but the differences were not statistically significant (*p* > 0.05). There were no cases of permanent recurrent laryngeal never injury or hypoparathyroidism in each group. Nevertheless, four patients experienced temporary recurrent laryngeal never palsy (1 in experiment group and 3 in conventional group): got they recovered within 3 months. No complications, such as subcutaneous fluid, tracheal injury, esophageal injury, mental nerve palsy, or infection, were found in each group postoperatively.

### Cosmetic outcome

**Figure 2:**
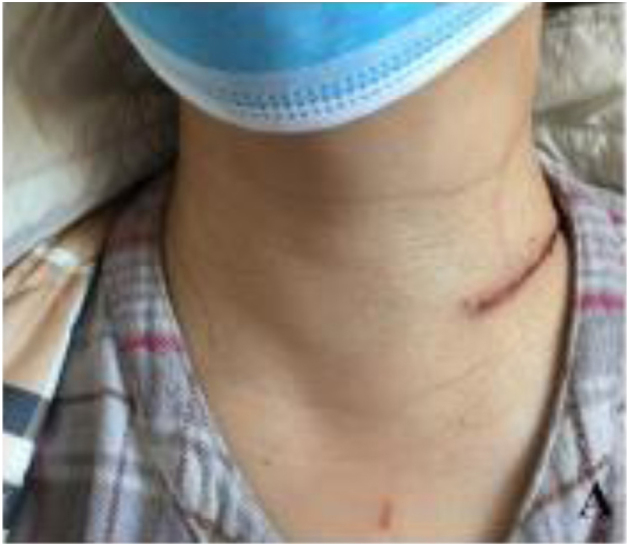
The appearance of the incision 3-days after the operation is demonstrated.

**Figure 3:**
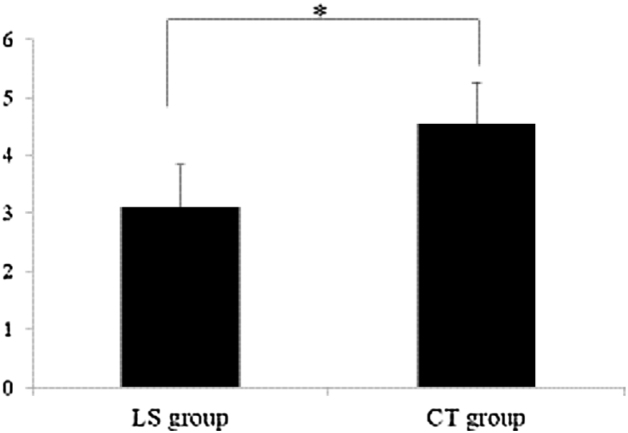
A statistically significant difference in postoperative pain, *p < 0.05.

All patients completed postoperative follow-up visits at third months. The cosmetic score of the incision in the experiment group (7.76 ± 1.87) was significantly higher than that in the conventional group (6.77 ± 2.15), and the difference was statistically significant (*p* < 0.05), Fig. 2 and Table 2 shows the picture of incisional appearance 3 months after the operation. The mean pain score postoperatively in the LS group was lower than that in the CT group when pain scores were measured on the 10-point VAS (3.12 ± 0. 45 vs. 4.55 ± 0. 49, *p* < 0.05), (Fig. 3).

**Table 2:** Cosmetic effect scores of each group.

Group	Number	Fair	Medium	Good	Scores
LS group	90	4	15	71	7.76 ± 1.87
CT group	90	10	22	58	6.77 ± 2.15
*t*	3.288				
*p*	***0.001***				

LS, lateral supraclavicular; CT, conventional transcervical; *t*, Independent sample t test. The greater the t value, the more significant the difference.

## Discussion

Surgical incisions and wounds have always been a source of worry for patients, and acceptable wound cosmetology has become a focus for thyroid surgeons. With technical advancement, thyroid procedures transitioned from conventional to video-assisted thyroidectomy and recently, to robot-assisted approaches for better cosmetic outcome.^12^

However, there are problems in the application of the endoscope in the neck: (1) one of the limitations is that endoscopic surgery requires extensive dissections in the thoracic and neck region, which raises multiple concerns regarding postoperative pain or complications.^13^ (2) another limitation is mental nerve injury, which was usually described by a patient as a unilateral loss of sensitivity of the lower lip. The incidence of postoperative mental nerve injury in transoral endoscopic thyroidectomy was 2.3%.^14^ (3) endoscopic thyroidectomy may increase the risk of thyroid capsule and tumor tissue rupture, with possible cell seeding in the process, during which the thyroid gland was extracted through a small skin incision.^15^, ^16^ (4) the operative time in endoscopic thyroidectomy is significantly longer than that in open surgery because such procedures require port placement and flap dissection. (5) carbon dioxide insufflations in the neck may cause adverse effect on hemodynamic and blood gas levels, such as hypercarbia, acidosis, decreased mean arterial pressure and central venous pressure.^17^ (6) transoral incision is categorized as a clean/contaminated wound and may turn a sterile incision into contaminated incision which can produce higher risk of infection.^18^ Finally, endoscopic thyroidectomy is still more expensive and requires a longer learning curve for surgeon.^19^

Since 2011, Chen^8^ had attempted to perform thyroid surgery via lateral supraclavicular incision to access thyroidectomy which was mainly applied in thyroid benign nodule surgery. There are still not many studies to confirm that the incision is a feasible and safe approach for resection of thyroid carcinoma. Clinical data from thyroid carcinoma were recorded for two groups. In this study, the results showed that there was no significant difference between the two groups in the characteristics of patients, intraoperative bleeding, operative time, postoperative total drainage, postoperative pain, central lymph node clearance, subcutaneous hydrops, tracheal injury, esophageal injury, infection, and other complications. These results suggest that both methods are safe and effective. The incidence of RLN injury and hypoparathyroidism are not significantly increased, possibly because of meticulous dissection. However, lateral supraclavicular incisions result in better cosmetic outcomes than conventional transcervical incisions. The favorable cosmetic effects of the lateral supraclavicular incision are due to: (1) ligation of the anterior jugular vein and transversal resection of strap muscles can be avoided during the operation, thereby preventing the postoperative disturbance of venous return and subscapular scar formation^20^; (2) cervical movements in the lateral part above the clavicle are much gentler and less frequent than the middle region; and (3) the pre-tracheal fascia and the potential tissue spaces are undisturbed.^20^

It is reasonable to question whether the completeness of the operation by lateral supraclavicular incision is comparable with that achieved with the traditional transcervical approach. This question can be answered with the data available from the evaluation of the completeness of unilateral thyroidectomy and central compartment lymph node dissection. Postoperative ultrasonography showed no residual thyroid tissue or the central neck lymph node (data not showed).^15^

Cumulatively, these results indirectly confirm that the two incisions are equally effective in the treatment of unilateral thyroid carcinoma. However, this study was associated with some limitations. First, this incision is only suitable for unilateral thyroid carcinoma; it is surgically difficult to deal with contralateral lesions in the same incision. Secondly, the study included a relatively small sample size and short-term follow-up. A larger data about evaluating the prognosis of thyroid carcinoma patients operated with this incision should be explored in larger studies that include longer follow-ups and an increased number of patients.

## Conclusion

Our study suggested that the lateral supraclavicular incision is a safe and practical approach for thyroidectomy. It provides satisfactory cosmetic outcomes compared with conventional transcervical incision. This technique can be easily performed by most of the surgeons who have traditional thyroidectomy experience, requiring a short learning curve.

## Ethical review

The research accepts the approval of the hospital's ethics committee (KY2020216).

## Conflicts of interest

The authors declare no conflicts of interest.
